# Beyond Ejection Fraction: Inflammatory Risk and Mortality in Heart Failure

**DOI:** 10.3390/clinpract16100175

**Published:** 2026-09-22

**Authors:** Bozhidar Krastev, Natalia Spasova, Desislava Somleva, Neli Georgieva, Slavi Yakov, Elena Kinova, Assen Goudev

**Affiliations:** 1 Department of Emergency Medicine, Medical University of Sofia, 1527 Sofia, Bulgaria; spasova.natalia@gmail.com (N.S.); desisomleva@abv.bg (D.S.); slavi_yakov93@abv.bg (S.Y.); kinova.e@abv.bg (E.K.); agoudev@abv.bg (A.G.); 2 Clinic of Cardiology, University Hospital “Tsaritsa Yoanna-ISUL”, 8 Byalo More Str., 1527 Sofia, Bulgaria; ngeorgieva87@gmail.com

**Keywords:** heart failure, systemic immune-inflammation index, all-cause mortality, prognosis, NT-proBNP

## Abstract

**Background:** Inflammation is increasingly recognized as an important contributor to the progression of heart failure and may partly explain differences in prognosis beyond conventional clinical characteristics. Simple laboratory-based inflammatory indices may offer additional prognostic information, but their value across different heart failure phenotypes remains uncertain. **Objectives:** To investigate the association between the systemic immune-inflammation index and recorded all-cause mortality across the spectrum of heart failure and to evaluate its incremental prognostic value beyond conventional clinical and laboratory variables. **Patients and Methods:** We evaluated the association between the systemic immune-inflammation index (SII) and recorded all-cause mortality in patients with heart failure. Multivariable logistic regression models included age, sex, heart failure phenotype, cardiovascular comorbidities, NT-proBNP, renal function, and hemoglobin. Model discrimination and calibration were assessed, and additional analyses evaluated multicollinearity, interaction with heart failure phenotype, and the functional form of the association. **Results:** Among 637 patients, mortality status was available for 634, of whom 165 died (26.0%). After multivariable adjustment, each doubling of SII was associated with higher odds of recorded all-cause mortality (OR 1.42, 95% CI 1.19–1.70; *p* < 0.001). Previous myocardial infarction, atrial fibrillation, and NT-proBNP also remained independently associated with mortality, whereas LVEF phenotype was not. No significant interaction was observed between SII and heart failure phenotype. Adding SII improved overall model fit (likelihood-ratio χ^2^ = 16.17; *p* < 0.001) and increased the AUC from 0.710 to 0.732, although the difference in discrimination was not statistically significant (DeLong *p* = 0.076). There was no convincing evidence of marked non-linearity. **Conclusions:** A higher systemic inflammatory burden was independently associated with recorded all-cause mortality across the heart failure spectrum. This readily available marker may provide complementary prognostic information, but its incremental value appears modest. External validation is needed before it can be considered for routine risk assessment.

## 1. Introduction

Heart failure remains a major challenge in contemporary cardiovascular medicine. It affects more than 60 million people worldwide, and its prevalence increases markedly with age [1,2]. As populations grow older and survival after myocardial infarction and other cardiovascular diseases improves, the number of people living with heart failure is expected to rise. Recurrent hospitalizations, long-term treatment, functional decline, and the need for multidisciplinary care place a substantial and growing burden on patients, families, and healthcare systems [2,3].

The treatment of heart failure has largely been shaped by the neurohormonal model, and inhibition of the renin–angiotensin–aldosterone and sympathetic nervous systems has improved outcomes, particularly in patients with reduced ejection fraction. However, heart failure remains a complex and heterogeneous syndrome that cannot be explained by neurohormonal activation alone. Patients with apparently similar clinical characteristics and left ventricular ejection fraction may follow very different clinical courses, indicating that other biological pathways contribute to disease progression.

Inflammation is one such pathway. Experimental and clinical evidence suggests that systemic and myocardial inflammation may contribute to endothelial dysfunction, adverse remodelling, fibrosis, impaired cardiomyocyte function, and multiorgan involvement in heart failure [4,5,6]. The inflammatory response is closely linked to common comorbidities, including chronic kidney disease, obesity, diabetes, coronary artery disease, and atrial fibrillation, although its importance is unlikely to be the same in every patient or across all heart failure phenotypes [5,6].

Interest in inflammation as a therapeutic target has also reached clinical trials. The HERMES trial is evaluating whether inhibition of interleukin-6 with ziltivekimab can improve outcomes in patients with mildly reduced or preserved ejection fraction and evidence of systemic inflammation [7]. Other ongoing studies, including ATHENA and POWER-HF, reflect the broader effort to understand heart failure beyond the traditional haemodynamic and neurohormonal framework and to identify biologically distinct groups that may benefit from targeted approaches. At present, however, inflammation remains a promising and actively investigated pathway rather than an established therapeutic target across the full spectrum of heart failure.

There is also a practical need for simple markers that reflect inflammatory activity in routine clinical practice. Many specialized inflammatory biomarkers are costly or not routinely measured, whereas the systemic immune-inflammation index (SII) can be calculated from platelet, neutrophil, and lymphocyte counts obtained from a standard complete blood count. By combining these three cellular components, SII provides an integrated estimate of the balance between systemic inflammation and immune regulation. Its low cost and wide availability make it an attractive candidate for clinical risk assessment.

Previous observational studies have linked higher SII values to adverse outcomes in selected populations with heart failure. Tang et al. reported an association between SII and short-term mortality in critically ill patients with congestive heart failure [8], while Wang et al. found prognostic value in patients with advanced chronic heart failure and renal dysfunction [9]. However, the available evidence remains limited. It is unclear whether the association between SII and mortality is consistent across LVEF-defined heart failure phenotypes or whether SII provides information beyond established clinical characteristics and routinely used laboratory markers.

The present study therefore aimed to evaluate the association between SII and recorded all-cause mortality in patients with heart failure. We also examined whether this association differed across heart failure phenotypes defined by left ventricular ejection fraction and whether SII provided incremental prognostic information beyond established clinical and laboratory variables.

## 2. Materials and Methods

### 2.1. Study Design and Population

This retrospective observational study included patients admitted with exacerbated or decompensated chronic heart failure to the Cardiology Clinic of University Hospital “Tsaritsa Yoanna–ISUL” between September 2023 and October 2025.

All patients were admitted with symptoms suggestive of worsening heart failure, most commonly dyspnoea and fatigue occurring at varying levels of physical activity. The diagnosis of exacerbated or decompensated chronic heart failure was established by the treating cardiology team on the basis of the clinical presentation, physical examination, laboratory findings, including NT-proBNP, and instrumental investigations, including echocardiography. All patients received diuretic therapy during the index hospitalization according to their clinical condition. Diagnostic and treatment data were obtained retrospectively from the hospital records and were not independently reassessed using a separate study-specific adjudication protocol.

During the study period, 1284 patients were hospitalized with exacerbated or decompensated chronic heart failure. NT-proBNP was measured as part of routine clinical practice rather than according to a predefined study protocol. Eligibility required the availability of NT-proBNP, left ventricular ejection fraction, and complete blood count measurements sufficient to calculate SII, together with fulfilment of the other inclusion and exclusion criteria. Eligibility did not depend on the NT-proBNP concentration, and no NT-proBNP cut-off was applied. Overall, 637 patients met all eligibility criteria and were included in the study cohort. Baseline data were collected only for the eligible cohort, and comparable clinical information was not systematically available for the remaining patients. Demographic data, cardiovascular risk factors, comorbidities, left ventricular ejection fraction, laboratory results, and pre-admission treatment were collected from the medical records. Collected laboratory variables included NT-proBNP, C-reactive protein, creatinine, hemoglobin, platelet count, neutrophil count, and lymphocyte count. The systemic immune-inflammation index was calculated as platelet count multiplied by neutrophil count and divided by lymphocyte count.

The first available laboratory values during the hospitalization were used as baseline measurements. In most patients, blood samples were obtained at admission, before treatment was started or intensified. However, because of the retrospective design, the exact timing of sampling could not be confirmed in every case. Therefore, the earliest available values were accepted as baseline.

Estimated glomerular filtration rate (eGFR) was calculated using the Modification of Diet in Renal Disease (MDRD) equation for the primary analysis. Left ventricular ejection fraction was assessed during the index hospitalization, usually at or shortly after admission.

Information on treatment before admission was also collected and included loop diuretics, angiotensin-converting enzyme inhibitors or angiotensin receptor blockers, beta-blockers, mineralocorticoid receptor antagonists, angiotensin receptor–neprilysin inhibitors, sodium–glucose cotransporter 2 inhibitors, and statins.

To minimize potential confounding of inflammatory markers, patients with active infection, ongoing malignancy, autoimmune disease, or ongoing systemic corticosteroid therapy were excluded from the analysis.

Mortality outcome could not be determined in three patients. Therefore, all 637 patients were included in the descriptive analyses, while analyses involving mortality were performed in the 634 patients with available outcome data.

### 2.2. Outcome Definition

The primary outcome was recorded all-cause mortality at the time of data extraction. Mortality status was ascertained through the National Health Insurance Fund database and hospital electronic medical records, which provides nationwide coverage of registered deaths. Exact dates of death were not available; therefore, mortality was analyzed as a binary outcome using logistic regression rather than time-to-event methods. Because patients entered the cohort over an extended inclusion period, the duration of observation differed between individuals. Accordingly, the reported odds ratios should be interpreted as associations with recorded mortality status rather than as estimates of time-dependent risk. Mortality status could not be determined for three patients, who were excluded from the outcome analyses. No additional patients were lost to follow-up.

### 2.3. Heart Failure Phenotypes

Patients were grouped according to the LVEF measured during the index hospitalization, using the European Society of Cardiology classification. HFrEF was defined as LVEF ≤ 40%, HFmrEF as LVEF 41–49%, and HFpEF as LVEF ≥ 50%.

### 2.4. Statistical Analysis

Statistical analyses were conducted using IBM SPSS Statistics, version 23.0 (IBM Corp., Armonk, NY, USA). The DeLong test for comparing correlated AUCs was performed using custom Python code (Python 3.13.15) executed within SPSS through IBM SPSS Statistics–Essentials for Python (Python Integration Plug-in). The code used the SPSS spssdata interface and the Python standard library; no additional third-party statistical package was required. Continuous variables were assessed for normality using the Shapiro–Wilk test and are presented as mean ± standard deviation or median (interquartile range), as appropriate. Categorical variables are presented as frequencies and percentages. Comparisons among the three heart failure phenotypes were performed using one-way analysis of variance (ANOVA) or the Kruskal–Wallis test, as appropriate, followed by Dunn’s post hoc test for pairwise comparisons after significant Kruskal–Wallis tests. Categorical variables were compared using the chi-square test.

The primary outcome was recorded all-cause mortality. Logistic regression analysis was used to identify factors associated with mortality. A multivariable clinical model included age, sex, left ventricular ejection fraction (LVEF) phenotype, coronary artery disease, previous myocardial infarction, stroke, diabetes mellitus, arterial hypertension, obesity, atrial fibrillation, and dyslipidemia. Age was entered as a continuous variable, with the odds ratio reported per 10-year increase. The LVEF phenotype was represented by two indicator variables, with HFpEF as the reference category, while the remaining clinical variables were entered as binary variables. All clinical variables were entered simultaneously into the model; no automated stepwise selection was used.

A second model also included N-terminal pro-B-type natriuretic peptide (NT-proBNP), systemic immune-inflammation index (SII), estimated glomerular filtration rate (eGFR), and hemoglobin. Variables in both models were selected because of their established or biologically plausible relevance to prognosis in heart failure rather than on the basis of univariable statistical significance; no univariable *p*-value threshold was applied. NT-proBNP and SII were analyzed on the log_2_ scale, and odds ratios are reported per doubling of each biomarker. eGFR was modelled per 10 mL/min/1.73 m^2^ increase, and hemoglobin per 10 g/L increase. To assess whether the choice of equation affected the results, we recalculated eGFR using the 2021 CKD-EPI creatinine equation and repeated the fully adjusted model with CKD-EPI eGFR in place of MDRD eGFR.

The functional form of the association between SII and recorded all-cause mortality was further examined using restricted cubic splines within the fully adjusted model. The spline basis terms were generated using custom SPSS syntax, with four knots placed at the 5th, 35th, 65th, and 95th percentiles of log_2_-transformed SII (8.3393, 9.4413, 10.3665, and 11.9475, respectively). The resulting three spline terms were entered into the standard binary logistic regression procedure, with the median SII value (915.4) used as the reference. The overall association was evaluated by comparing the spline model with the corresponding model without SII, while non-linearity was assessed by comparing the spline model with the model containing SII as a linear log_2_-transformed variable, using likelihood-ratio tests. To evaluate the incremental prognostic value of SII, the clinical model including NT-proBNP was compared with the model that also included SII using likelihood-ratio testing. Model discrimination was assessed by the area under the receiver operating characteristic curve (AUC), and AUCs were compared using the DeLong test. The individual predicted probabilities generated by each model were used for ROC curve analysis. Model fit was additionally compared using the Akaike information criterion (AIC), Bayesian information criterion (BIC), and the Brier score.

Model calibration was evaluated using the Hosmer–Lemeshow goodness-of-fit test, the Brier score, and calibration slope with bootstrap internal validation. Multicollinearity was assessed using variance inflation factors (VIFs), tolerance values, and the condition index.

Potential effect modification by LVEF phenotype was evaluated by introducing interaction terms between phenotype and NT-proBNP, SII, eGFR, and hemoglobin into the fully adjusted model. Interaction models were compared with the corresponding models without interaction terms using likelihood-ratio tests.

To assess the possible influence of differences in observation time, a sensitivity analysis was performed by repeating the fully adjusted model in patients with at least 12 months of potential observation. Because mortality status was assessed on 1 July 2026, this analysis was restricted to patients whose index hospitalization occurred on or before 1 July 2025.

C-reactive protein was not included in the primary multivariable model because values were missing for 61 of the 637 patients (9.6%). Its inclusion would have reduced the complete-case sample for the outcome analysis from 634 to 573 patients. As a sensitivity analysis, the fully adjusted model was repeated among these 573 patients, with log_2_-transformed CRP added as an additional covariate. A two-sided *p*-value < 0.05 was considered statistically significant.

## 3. Results

Baseline characteristics according to LVEF phenotype are presented in Table 1.

A total of 637 patients were included: 182 with HFrEF, 98 with HFmrEF, and 357 with HFpEF. Patients with HFmrEF were slightly older, while men were more common in the HFrEF and HFmrEF groups. Previous myocardial infarction was most frequent in HFrEF, and atrial fibrillation was most common in HFmrEF.

Most other comorbidities were similarly distributed across the three phenotypes. Pre-admission treatment also differed across phenotypes, with ARNI used more frequently in HFrEF and ACE inhibitor or ARB use more frequently in HFpEF.

Recorded all-cause mortality was observed in 165 of 634 patients (26.0%) and was numerically most frequent in HFmrEF, but the difference between the LVEF groups was not statistically significant.

Age and NT-proBNP differed significantly across LVEF phenotypes. Patients with HFrEF were younger than those with HFmrEF and HFpEF, while NT-proBNP levels were significantly higher in HFrEF than in both other groups. Dunn’s test confirmed these differences after Holm correction. No significant differences were observed in eGFR, SII, or hemoglobin (Table 2).

Abbreviations: eGFR—estimated glomerular filtration rate; HFmrEF—heart failure with mildly reduced ejection fraction; HFpEF—heart failure with preserved ejection fraction; HFrEF—heart failure with reduced ejection fraction; LVEF—left ventricular ejection fraction; NT-proBNP—N-terminal pro-B-type natriuretic peptide; SII—systemic immune-inflammation index.

In the multivariable clinical model, older age, previous myocardial infarction, and atrial fibrillation were independently associated with recorded all-cause mortality (Table 3). Each 10-year increase in age was associated with 30% higher odds of mortality (OR 1.30, 95% CI 1.07–1.58; *p* = 0.007). Previous myocardial infarction was associated with almost threefold higher odds of mortality (OR 2.89, 95% CI 1.60–5.23; *p* < 0.001), while atrial fibrillation was associated with 74% higher odds (OR 1.74, 95% CI 1.18–2.56; *p* = 0.006). Arterial hypertension was associated with lower odds of recorded mortality (OR 0.60, 95% CI 0.37–0.96; *p* = 0.034), although this association was no longer statistically significant after laboratory variables were added.

In the fully adjusted clinical and laboratory model, previous myocardial infarction, atrial fibrillation, NT-proBNP, and SII were independently associated with recorded all-cause mortality (Table 4). A history of myocardial infarction was associated with almost threefold higher odds of mortality (OR 2.77, 95% CI 1.50–5.12; *p* = 0.001), while atrial fibrillation was associated with a 62% increase in the odds (OR 1.62, 95% CI 1.07–2.46; *p* = 0.022). Each doubling of NT-proBNP was associated with 23% higher odds of mortality (OR 1.23, 95% CI 1.08–1.40; *p* = 0.001), and each doubling of SII with 42% higher odds (OR 1.42, 95% CI 1.19–1.70; *p* < 0.001). eGFR, hemoglobin, age, sex, and LVEF phenotype were not independently associated with mortality in the fully adjusted model.

The sensitivity analysis restricted to patients with at least 12 months of potential observation included 549 patients, of whom 147 had died. The results were consistent with those of the main analysis. The association between SII and recorded all-cause mortality remained statistically significant, with an OR of 1.48 per doubling (95% CI 1.22–1.80; *p* < 0.001), compared with an OR of 1.42 (95% CI 1.19–1.70; *p* < 0.001) in the main analysis. The complete results are presented in Supplementary Table S6.

Recalculation of eGFR using the 2021 CKD-EPI creatinine equation did not materially alter the results. SII remained independently associated with recorded all-cause mortality (OR per doubling 1.43, 95% CI 1.19–1.71; *p* < 0.001), whereas CKD-EPI-based eGFR was not independently associated with the outcome (OR per 10 mL/min/1.73 m^2^ increase 0.98, 95% CI 0.90–1.06; *p* = 0.582). The complete results are presented in Supplementary Table S7.

In an additional sensitivity analysis including 573 complete cases (157 deaths), the association between SII and recorded all-cause mortality remained statistically significant after additional adjustment for CRP (OR per doubling 1.26, 95% CI 1.04–1.53; *p* = 0.021). CRP was also significantly associated with recorded all-cause mortality (OR per doubling 1.17, 95% CI 1.04–1.32; *p* = 0.010). The complete results are presented in Supplementary Table S8. Model calibration and multicollinearity were assessed in supplementary analyses. The Hosmer–Lemeshow test did not indicate significant lack of fit (χ^2^ = 6.66, df = 8; *p* = 0.573). The apparent Brier score was 0.164, while the bootstrap-corrected Brier score was 0.174. After internal validation, the bootstrap-corrected calibration slope was 0.82, suggesting a modest degree of model overfitting.

The addition of SII to the clinical model including NT-proBNP significantly improved overall model fit according to the likelihood-ratio test (χ^2^ = 16.17, df = 1; *p* < 0.001), with lower AIC, BIC, and Brier score values (Supplementary Table S4). The AUC increased from 0.710 to 0.732, although the improvement in discrimination did not reach statistical significance (DeLong *p* = 0.076; Figure 1).

No relevant multicollinearity was identified among the predictors included in the final model. Detailed calibration and multicollinearity diagnostics are presented in Supplementary Tables S1 and S2.

No statistically significant interactions were detected between LVEF phenotype and NT-proBNP, SII, eGFR, or hemoglobin (all interaction *p* > 0.05). Thus, the available data did not provide evidence that these associations differed across LVEF phenotypes. Detailed interaction analyses are provided in Supplementary Table S3.

Restricted cubic spline analysis demonstrated a significant overall association between SII and recorded all-cause mortality after multivariable adjustment (likelihood-ratio χ^2^ = 21.37, df = 3; *p* < 0.001). The test for non-linearity was at the prespecified significance threshold but did not fall below it (χ^2^ = 5.98, df = 2; *p* = 0.050), providing no clear evidence against the log-linear specification used in the primary model (Figure 2).

## 4. Discussion

In this cohort of patients hospitalized with heart failure, higher SII was independently associated with recorded all-cause mortality after adjustment for clinical characteristics, cardiovascular comorbidities, NT-proBNP, renal function, and hemoglobin. Each doubling of SII was associated with a 42% increase in the odds of recorded mortality. Previous myocardial infarction, atrial fibrillation, and NT-proBNP were also independently associated with the outcome, whereas LVEF phenotype was not. The association between SII and mortality was similar across HFrEF, HFmrEF, and HFpEF.

These findings support the view of heart failure as a systemic syndrome that is not determined by ventricular systolic function alone. Inflammatory activation interacts with haemodynamic, neurohormonal, metabolic, vascular, and cardiorenal pathways and may contribute to myocardial injury, fibrosis, adverse remodelling, and disease progression [10,11]. Such mechanisms are especially relevant to HFpEF, where ageing and comorbidity-related inflammation have been linked to endothelial dysfunction and myocardial structural changes [12], although our results do not indicate that the prognostic association of SII is limited to this phenotype.

SII may capture part of this inflammatory burden using data already available from a routine complete blood count. It combines neutrophil, platelet, and lymphocyte counts, reflecting related but distinct components of the immune response. Neutrophilia is associated with innate immune activation, platelets contribute to inflammatory and thrombotic signalling, and lymphopenia may reflect physiological stress or impaired adaptive immune regulation. SII therefore offers a broader estimate of systemic inflammatory activity than an individual blood-cell count, but it remains non-specific and may be affected by infection, malignancy, inflammatory disorders, medication, and other comorbidities.

SII has also been studied outside heart failure, including calcific severe aortic stenosis, the no-reflow phenomenon after primary percutaneous coronary intervention, and HFrEF treated with an implantable cardioverter-defibrillator [13,14,15]. Together, these studies suggest that SII may reflect inflammatory and immune activation across cardiovascular conditions, although its clinical meaning depends on the population and outcome studied.

Our results are consistent with previous observational studies in heart failure. Tang et al. linked higher SII to short-term mortality in critically ill patients with congestive heart failure [8]. Wang et al. examined patients with advanced chronic heart failure and renal dysfunction [9], Yuan et al. studied a broader heart failure population [16], and Özmen et al. reported an association with one-year all-cause mortality in HFrEF, although SII alone had modest discriminatory performance [17]. Despite differences in patient selection, outcomes, and methods, these studies generally associate higher SII with poorer prognosis.

Other composite inflammatory indicators are also under investigation. Yang et al. compared several such indices in relation to short-term outcomes, showing that SII is one of several blood cell-based measures rather than an established stand-alone prognostic tool [18]. Our study focused on SII and cannot determine whether it performs better than alternative indices.

A systematic review and meta-analysis of 15 retrospective studies involving 77,917 patients found that elevated SII was associated with higher mortality in heart failure, with a pooled risk ratio of 1.44, although between-study heterogeneity was substantial. Subgroup analyses did not show clear modification by ejection fraction [19]. These pooled data support the prognostic relevance of SII, but differences in thresholds, populations, and outcomes, together with the retrospective design of the included studies, limit direct clinical application.

The present study extends this evidence by including HFrEF, HFmrEF, and HFpEF within the same hospitalized cohort. Earlier studies often focused on critically ill patients, advanced disease, renal dysfunction, or reduced ejection fraction. Our results therefore complement the HFrEF findings of Özmen et al. and emerging phenotype-specific evidence in HFpEF.

In a recent HFpEF cohort, Zhu et al. found that SII was associated with cardiovascular mortality and heart failure rehospitalization and outperformed several conventional inflammatory indices for these outcomes, but it was not independently associated with all-cause mortality [20]. This difference suggests that the prognostic value of SII may depend on the endpoint. Our study assessed recorded all-cause mortality and could not distinguish cardiovascular from non-cardiovascular deaths.

The absence of a significant interaction between SII and heart failure phenotype suggests that the association with mortality was not confined to one LVEF category. Inflammatory activation may contribute to adverse remodelling in HFrEF, while in HFpEF it is closely linked to ageing and comorbidities such as obesity, diabetes, hypertension, renal dysfunction, and atrial fibrillation [10,11,12]. SII may therefore reflect processes shared across the heart failure spectrum.

This is consistent with the similar SII levels across the three LVEF groups and the lack of an independent association between LVEF phenotype and mortality in the multivariable models. It does not imply that LVEF lacks clinical or prognostic importance. LVEF remains central to heart failure classification and treatment selection, but patients with similar ejection fractions may differ substantially in age, ischaemic heart disease, atrial fibrillation, renal function, natriuretic peptide concentration, inflammatory status, and comorbidity burden. The interaction analysis also warrants caution because the HFmrEF subgroup was relatively small, and more subtle differences between phenotypes may not have been detected.

Previous myocardial infarction and atrial fibrillation remained independently associated with recorded mortality. Previous myocardial infarction may identify a greater burden of ischaemic injury, scar formation, and adverse remodelling, whereas atrial fibrillation may reflect more advanced structural heart disease, elevated filling pressures, atrial remodelling, and comorbidity. Both may therefore add prognostic information beyond LVEF.

The inverse association between arterial hypertension and recorded mortality in the clinical model should not be interpreted as evidence of a protective effect. Patients without a documented history of hypertension may have included individuals with more advanced heart failure, lower blood pressure, or less haemodynamic reserve, and hypertension may also have been under-recorded in some patients. The association was attenuated and no longer statistically significant after the laboratory variables were added, suggesting that it may have reflected differences in disease severity or other patient characteristics rather than an independent effect of hypertension. This finding should therefore be interpreted with caution.

The association between NT-proBNP and mortality was expected given its established role as a marker of myocardial wall stress and heart failure severity. SII remained independently associated with mortality after NT-proBNP was included, suggesting that the two biomarkers may capture different aspects of risk: NT-proBNP mainly reflects haemodynamic and myocardial stress, whereas SII may add information on systemic inflammatory and immune activity.

Adding SII produced a modest increase in AUC that did not reach statistical significance, but the extended model had better overall fit, lower AIC and BIC values, and a small improvement in the Brier score. These findings support the use of SII, if validated, as a complementary marker rather than a replacement for established prognostic variables.

Restricted cubic spline analysis provided no clear evidence that a non-linear model described the association better than the log-linear form used in the main analysis. Zhang et al. also analyzed log-transformed SII continuously and used restricted cubic splines in heart failure [21]. In that cohort, higher SII was associated with all-cause mortality but not independently with cardiovascular mortality, again suggesting that the observed relationship may depend partly on the outcome definition. Continuous modelling also avoids information loss and dependence on cut-off values that may not transfer between populations.

The model showed satisfactory apparent calibration, while bootstrap validation indicated some optimism, as expected in an internally developed model. Multicollinearity was low, making it unlikely that the SII association resulted from instability caused by strong correlations among predictors. These results support the internal consistency of the analysis but do not replace external validation.

The main practical advantage of SII is its availability: it can be calculated from a routine complete blood count without additional testing or cost. Its contribution in this cohort was modest, but it may offer an additional risk signal alongside clinical assessment, NT-proBNP, renal function, and other established markers. Current observational and pooled evidence supports further study but does not define a standardized threshold or a role in clinical decision-making [8,9,16,17,18,19,20]. Prognostic association also does not mean that lowering SII would improve outcomes, and experience with inflammatory pathways in heart failure shows that biological plausibility does not necessarily translate into effective anti-inflammatory treatment [10,11].

Several limitations should be considered. This was a retrospective, single-centre study, so causal conclusions cannot be drawn. Patients entered the cohort over an extended period and therefore had unequal observation times before mortality ascertainment. Exact dates of death were unavailable, preventing survival analysis; mortality was analyzed as a binary outcome, and the odds ratios should not be interpreted as hazard ratios or as risk estimates over a fixed follow-up period. Nationwide mortality ascertainment minimized loss to follow-up, and the sensitivity analysis restricted to patients with at least 12 months of potential observation produced similar estimates. Nevertheless, this analysis could not fully account for differences in individual observation time. Only all-cause mortality was available, and cardiovascular deaths could not be separated from other causes, which is relevant for a non-specific marker such as SII. The index was calculated from the earliest available laboratory results during hospitalization, but sampling in relation to treatment and clinical stabilization could not be standardized. A single value may reflect an acute or transient inflammatory response, and serial changes were not assessed. CRP was not included in the primary model because of missing data; however, the association between SII and recorded all-cause mortality remained statistically significant in the complete-case sensitivity analysis additionally adjusted for CRP.

Selection bias is possible because inclusion required available LVEF, NT-proBNP, and complete blood count data sufficient to calculate SII. NT-proBNP was measured according to routine clinical practice rather than a standardized study protocol, and patients who underwent biomarker testing may therefore have differed from those who did not. Because complete baseline information was not systematically collected for patients who did not meet the eligibility criteria, a reliable comparison between included and excluded patients could not be performed. Mortality status was unavailable for three otherwise eligible patients. External validation was not performed, and the relatively small HFmrEF subgroup limited the precision of phenotype-specific analyses. Despite these limitations, the study included patients across the LVEF spectrum and assessed SII alongside established clinical and laboratory predictors. The analysis included multivariable modelling, interaction testing, restricted cubic splines, measures of incremental model performance, calibration, bootstrap validation, and multicollinearity diagnostics.

## 5. Conclusions

Higher SII was independently associated with recorded all-cause mortality in patients hospitalized with heart failure, even after adjustment for clinical characteristics, NT-proBNP, renal function, and hemoglobin. The association was similar across HFrEF, HFmrEF, and HFpEF, with no significant interaction by heart failure phenotype. Adding SII improved overall model fit, but the gain in discrimination was modest. SII may be useful as an inexpensive additional marker of risk, although its value should be confirmed in external and prospective cohorts before it is used routinely in clinical practice.

Because exact dates and causes of death were unavailable, survival and cardiovascular mortality analyses could not be performed. The findings should therefore be interpreted as associations with recorded all-cause mortality status rather than as estimates of time-dependent or cause-specific risk.

## Figures and Tables

**Figure 1:**
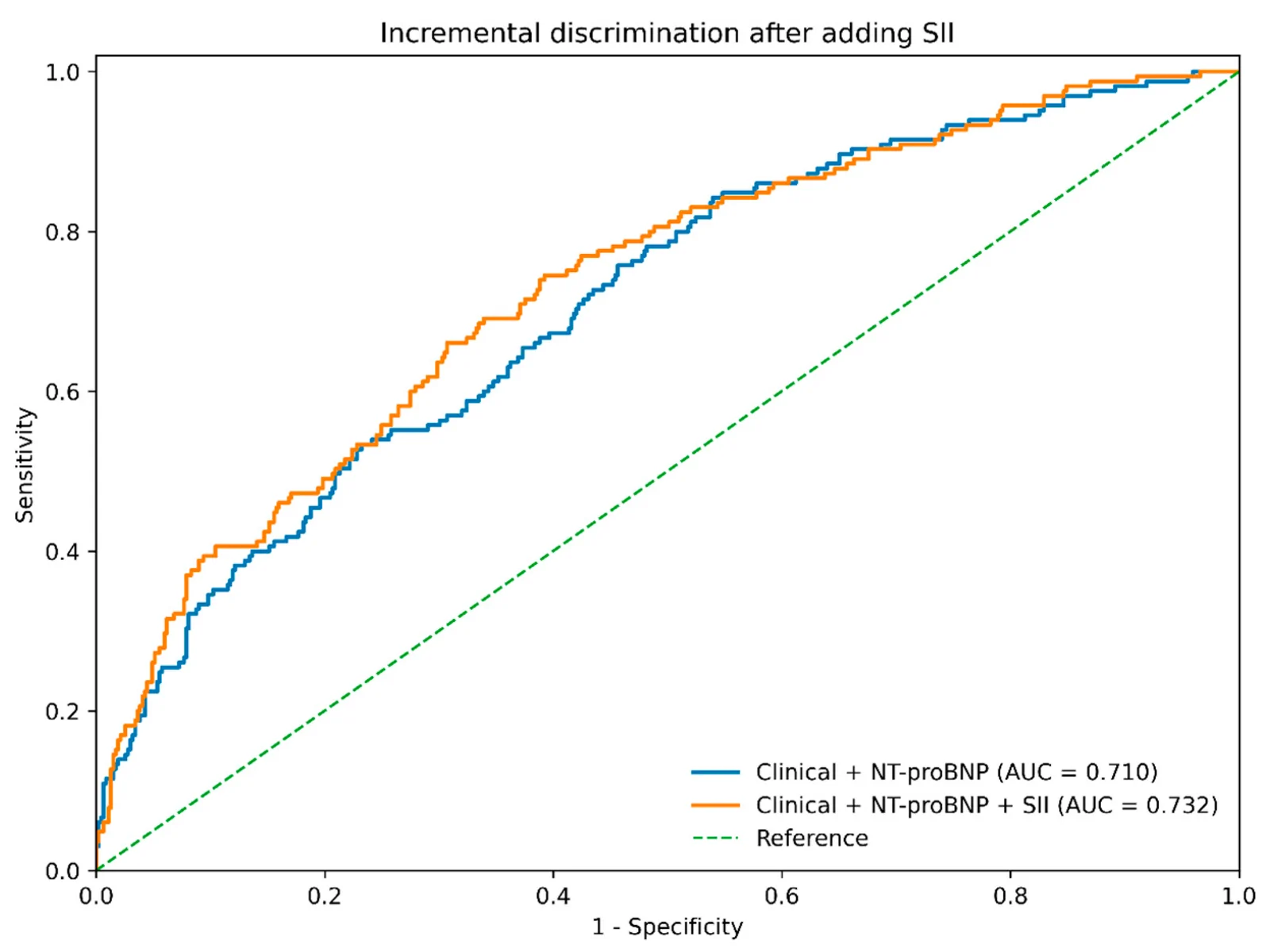
Receiver operating characteristic curves comparing the clinical model including NT-proBNP with the extended model additionally including SII. The addition of SII increased the AUC from 0.710 to 0.732; however, the difference in discrimination was not statistically significant according to the DeLong test (*p* = 0.076). SII significantly improved overall model fit according to the likelihood-ratio test (χ^2^ = 16.17, df = 1; *p* < 0.001).

**Figure 2:**
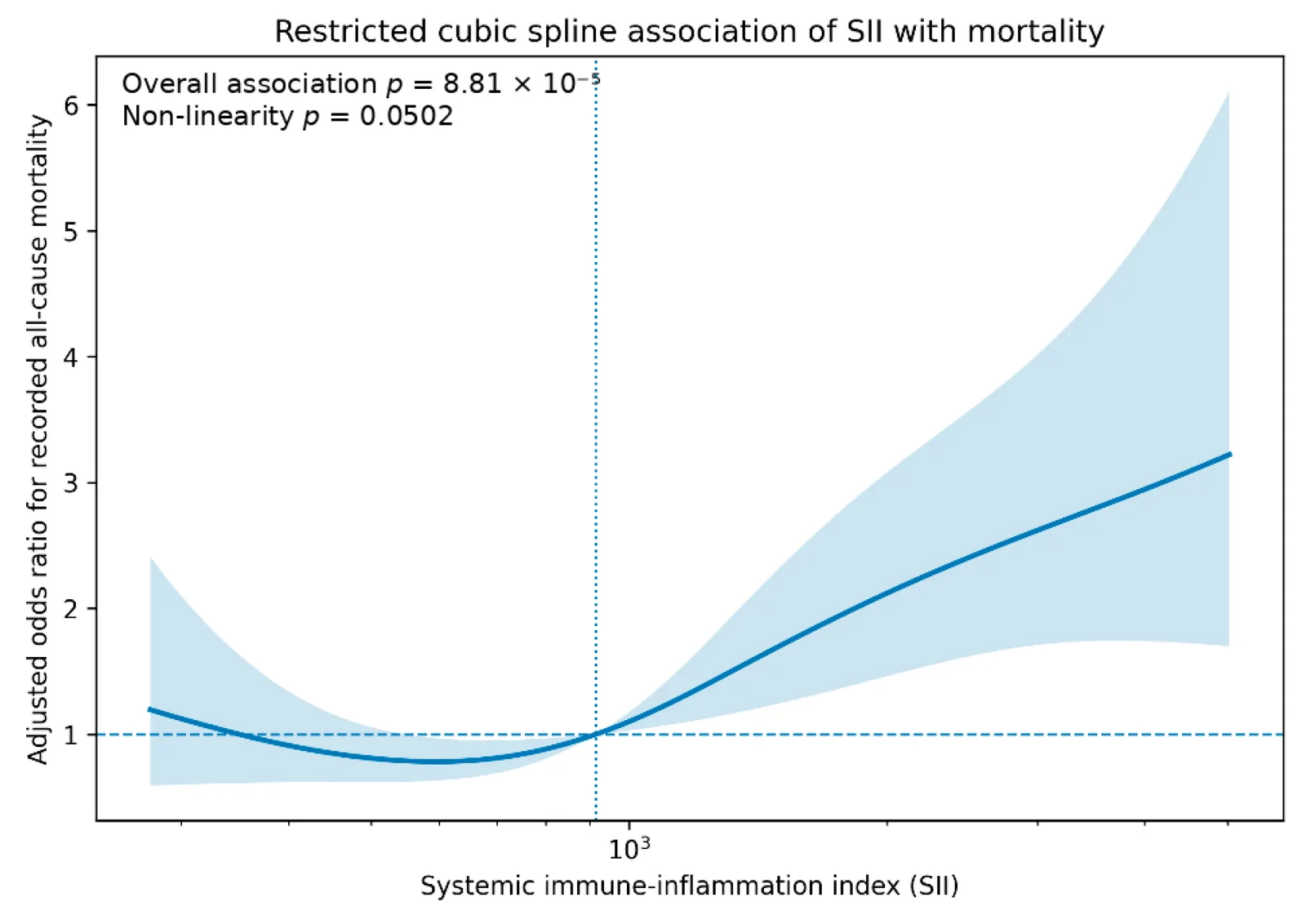
Adjusted association between SII and recorded all-cause mortality modelled using restricted cubic splines. The solid line shows the adjusted odds ratio and the shaded area shows the 95% confidence interval. The model was adjusted for clinical covariates and laboratory parameters. The horizontal dashed line indicates an odds ratio of 1.0, and the vertical line marks the median SII value used as the reference.

**Table 1:** Baseline characteristics according to LVEF phenotype.

Characteristic	Overall (*n* = 637)	HFrEF (*n* = 182)	HFmrEF (*n* = 98)	HFpEF (*n* = 357)	*p* Value
Demographics and clinical characteristics					
Age, years	75 [66–81]	71.5 [63.3–78.8]	77.0 [67.0–82.0]	75.0 [66–82]	0.004
Male sex	323 (50.7)	118 (64.8)	61 (62.2)	144 (40.3)	<0.001
LVEF, %	50 [40–57]	32 [25–38]	45 [45–47]	55 [51–60]	<0.001
Comorbidities					
Coronary artery disease	153 (24.0)	54 (29.7)	24 (24.5)	75 (21.0)	0.083
Previous myocardial infarction	87 (13.7)	41 (22.5)	11 (11.2)	35 (9.8)	<0.001
Previous stroke	93 (14.6)	29 (15.9)	11 (11.2)	53 (14.8)	0.556
Diabetes mellitus	216 (33.9)	62 (34.1)	26 (26.5)	128 (35.9)	0.225
Arterial hypertension	524 (82.3)	142 (78.0)	81 (82.7)	301 (84.3)	0.194
Obesity	66 (10.4)	13 (7.1)	13 (13.3)	40 (11.2)	0.203
Atrial fibrillation	294 (46.2)	88 (48.4)	55 (56.1)	151 (42.3)	0.041
Dyslipidemia	254 (39.9)	83 (45.6)	42 (42.9)	129 (36.1)	0.085
Pre-admission therapy					
Statin	224 (35.3)	69 (38.3)	33 (33.7)	122 (34.2)	0.595
ACE inhibitor/ARB	282 (44.4)	68 (37.6)	35 (35.7)	179 (50.3)	0.003
Beta-blocker	443 (69.8)	120 (66.3)	67 (68.4)	256 (71.9)	0.387
Mineralocorticoid receptor antagonist	207 (32.6)	60 (33.1)	38 (38.8)	109 (30.6)	0.307
Angiotensin receptor–neprilysin inhibitor	29 (4.6)	19 (10.5)	5 (5.1)	5 (1.4)	<0.001
Sodium–glucose cotransporter-2 inhibitor	185 (29.1)	55 (30.4)	31 (31.6)	99 (27.8)	0.692
Diuretic	367 (57.8)	96 (53.0)	58 (59.2)	213 (59.8)	0.307
Clinical outcome					
Recorded all-cause mortality	165 (26.0)	50 (27.8)	31 (31.6)	84 (23.6)	0.225

Data are presented as median [interquartile range] or n (%). Percentages are calculated using available data. *p* values were calculated using the Kruskal–Wallis test for continuous variables and the chi-square test for categorical variables. The outcome was available for 634 patients (165 events); three patients had missing outcome data. ACEI—angiotensin-converting enzyme inhibitor; ARB—angiotensin receptor blocker—ARNI—angiotensin receptor–neprilysin inhibitor; HFmrEF—heart failure with mildly reduced ejection fraction; HFpEF—heart failure with preserved ejection fraction; HFrEF—heart failure with reduced ejection fraction; LVEF—left ventricular ejection fraction. Mortality data were available for 180 patients with HFrEF, 98 with HFmrEF, and 356 with HFpEF.

**Table 2:** Selected clinical and laboratory parameters according to LVEF phenotype.

Parameter	HFrEF (*n* = 182)	HFmrEF (*n* = 98)	HFpEF (*n* = 357)	*p*-Value
Age, years	71.5 [63.3–78.8]	77.0 [67.0–82.0]	75.0 [66.0–82.0]	0.004
NT-proBNP, pg/mL	7871.9 [3910.1–16,010.3]	3567.0 [1210.0–7912.8]	2201.0 [791.0–5887.0]	<0.001
eGFR, mL/min/1.73 m^2^	55.4 [38.7–76.5]	56.5 [43.1–73.0]	57.0 [42.0–77.9]	0.646
SII	997.7 [590.0–1654.9]	833.6 [556.8–1430.4]	913.4 [572.6–1736.9]	0.328
Hemoglobin, g/L	133.0 [118.0–145.0]	128.5 [112.3–143.8]	129.0 [109.0–145.0]	0.155

Data are presented as a median [interquartile range]. Comparisons were performed using the Kruskal–Wallis test. Post hoc Dunn testing with Holm correction showed that patients with HFrEF were younger than those with HFmrEF (adjusted *p* = 0.029) and HFpEF (adjusted *p* = 0.006), with no difference between HFmrEF and HFpEF (adjusted *p* = 0.843). NT-proBNP was higher in HFrEF than in HFmrEF and HFpEF (both adjusted *p* < 0.001), while the difference between HFmrEF and HFpEF was not significant (adjusted *p* = 0.057).

**Table 3:** Multivariable analysis of recorded all-cause mortality: clinical model.

Variable	Adjusted OR	95% CI	*p*-Value
Age (per 10-year increase)	1.30	1.07–1.58	0.007
Male sex	1.00	0.66–1.49	0.981
HFrEF vs. HFpEF	1.16	0.74–1.81	0.514
HFmrEF vs. HFpEF	1.50	0.89–2.53	0.128
Coronary artery disease	0.85	0.51–1.42	0.534
Previous myocardial infarction	2.89	1.60–5.23	<0.001
Previous stroke	1.57	0.95–2.59	0.076
Diabetes mellitus	1.22	0.81–1.82	0.337
Arterial hypertension	0.60	0.37–0.96	0.034
Obesity	0.69	0.33–1.47	0.339
Atrial fibrillation	1.74	1.18–2.56	0.006
Dyslipidemia	0.69	0.46–1.05	0.084

OR—odds ratio; CI—confidence interval. Reference category: HFpEF.

**Table 4:** Multivariable analysis of recorded all-cause mortality: clinical and laboratory model.

Variable	Adjusted OR	95% CI	*p*-Value
Age, per 10-year increase	1.18	0.96–1.45	0.110
Male sex	1.02	0.67–1.55	0.932
HFrEF vs. HFpEF	0.81	0.49–1.34	0.411
HFmrEF vs. HFpEF	1.52	0.89–2.62	0.128
Coronary artery disease	0.92	0.54–1.55	0.749
Previous myocardial infarction	2.77	1.50–5.12	0.001
Previous stroke	1.62	0.96–2.74	0.069
Diabetes mellitus	1.12	0.74–1.72	0.590
Arterial hypertension	0.69	0.41–1.14	0.145
Obesity	0.64	0.29–1.42	0.274
Atrial fibrillation	1.62	1.07–2.46	0.022
Dyslipidemia	0.75	0.49–1.16	0.199
NT-proBNP, per doubling	1.23	1.08–1.40	0.001
SII, per doubling	1.42	1.19–1.70	<0.001
eGFR, per 10 mL/min/1.73 m^2^ increase	0.99	0.92–1.08	0.886
Hemoglobin, per 10 g/L increase	0.96	0.89–1.05	0.402

OR—odds ratio; CI—confidence interval; SII—systemic immune-inflammation index; eGFR—estimated glomerular filtration rate. Reference categories: female sex and HFpEF. NT-proBNP and SII were log_2_-transformed and are presented per doubling.

## Data Availability

The datasets used and analyzed during the current study are available from the corresponding author on reasonable request.

## Supplementary Materials

The following supporting information can be downloaded at: https://www.mdpi.com/article/10.3390/clinpract16100175/s1, Table S1. Calibration and internal validation of the clinical and laboratory model for recorded all-cause mortality. Table S2. Assessment of multicollinearity in the clinical and laboratory model. Table S3. Interaction analysis between laboratory parameters and LVEF phenotype for recorded all-cause mortality. Table S4. Exploratory incremental model performance after addition of SII. Table S5. Restricted cubic spline analysis of SII and recorded all-cause mortality. Table S6. Sensitivity analysis restricted to patients with at least 12 months of potential observation. Table S7. Sensitivity analysis using the 2021 CKD-EPI creatinine equation in the fully adjusted mortality model. Table S8. Sensitivity analysis including CRP in the fully adjusted mortality model. Figure S1. Adjusted restricted cubic spline association between SII and recorded all-cause mortality. The solid line shows the adjusted odds ratio and the shaded area the 95% confidence interval. The horizontal dashed line indicates OR = 1, and the vertical dotted line indicates the median SII reference value.

## Abbreviations

The following abbreviations are used in this manuscript:

ACEI	Angiotensin-converting enzyme inhibitor
AIC	Akaike information criterion
ARB	Angiotensin receptor blocker
ARNI	Angiotensin receptor–neprilysin inhibitor
AUC	Area under the receiver operating characteristic curve
BIC	Bayesian information criterion
CI	Confidence interval
CRP	C-reactive protein
eGFR	Estimated glomerular filtration rate
ESC	European Society of Cardiology
HF	Heart failure
HFmrEF	Heart failure with mildly reduced ejection fraction
HFpEF	Heart failure with preserved ejection fraction
HFrEF	Heart failure with reduced ejection fraction
LVEF	Left ventricular ejection fraction
MRA	Mineralocorticoid receptor antagonist
NT-proBNP	N-terminal pro-B-type natriuretic peptide
OR	Odds ratio
SII	Systemic immune-inflammation index
SGLT2	Sodium–glucose cotransporter 2
VIF	Variance inflation factor

## References

[1] Savarese G., Becher P.M., Lund L.H., Seferovic P., Rosano G.M.C., Coats A.J.S. (2023). Global burden of heart failure: A comprehensive and updated review of epidemiology. Cardiovasc. Res..

[2] Groenewegen A., Rutten F.H., Mosterd A., Hoes A.W. (2020). Epidemiology of heart failure. Eur. J. Heart Fail..

[3] Lesyuk W., Kriza C., Kolominsky-Rabas P. (2018). Cost-of-illness studies in heart failure: A systematic review 2004–2016. BMC Cardiovasc. Disord..

[4] Mann D.L. (2015). Innate immunity and the failing heart: The cytokine hypothesis revisited. Circ. Res..

[5] Murphy S.P., Kakkar R., McCarthy C.P., Januzzi J.L. (2020). Inflammation in heart failure: JACC state-of-the-art review. J. Am. Coll. Cardiol..

[6] Adamo L., Rocha-Resende C., Prabhu S.D., Mann D.L. (2020). Reappraising the role of inflammation in heart failure. Nat. Rev. Cardiol..

[7] Petrie M.C., Borlaug B.A., Buchholtz K., Ducharme A., Hvelplund A., Ping C.L.S., Mendieta G., Hardt-Lindberg S.Ø., Voors A.A., Ridker P.M. (2024). HERMES: Effects of ziltivekimab versus placebo on morbidity and mortality in patients with heart failure with mildly reduced or preserved ejection fraction and systemic inflammation. J. Card. Fail..

[8] Tang Y., Zeng X., Feng Y., Chen Q., Liu Z., Luo H., Zha L., Yu Z. (2021). Association of systemic immune-inflammation index with short-term mortality of congestive heart failure: A Retrospective Cohort Study. Front. Cardiovasc. Med..

[9] Wang J., Zhen Q., Yuan R., Guo J., Xu S., Lv Y., Xu Y., Lu Y., Gao J., Yu F. (2023). Systemic immune-inflammation index as a prognostic marker for advanced chronic heart failure with renal dysfunction. ESC Heart Fail..

[10] Hanna A., Frangogiannis N.G. (2020). Inflammatory cytokines and chemokines as therapeutic targets in heart failure. Cardiovasc. Drugs Ther..

[11] Boulet J., Sridhar V.S., Bouabdallaoui N., Tardif J.C., White M. (2024). Inflammation in heart failure: Pathophysiology and therapeutic strategies. Inflamm. Res..

[12] Segar M.W., Coulter S.A. (2023). Inflammatory pathways and their implications in heart failure with preserved ejection fraction. Tex. Heart Inst. J..

[13] Erdoğan M., Öztürk S., Kardeşler B., Yiğitbaşı M., Kasapkara H.A., Baştuğ S., Erdöl M.A., Bayram N.A., Akçay M., Durmaz T. (2021). The relationship between calcific severe aortic stenosis and systemic immune-inflammation index. Echocardiography.

[14] Esenboğa K., Kurtul A., Yamantürk Y.Y., Tan T.S., Tutar D.E. (2022). Systemic immune-inflammation index predicts no-reflow phenomenon after primary percutaneous coronary intervention. Acta Cardiol..

[15] Hayıroğlu M.Ç., Çınar T., Çinier G., Pay L., Yumurtaş A., Tezen O., Eren S., Kolak Z., Çetin T., Çiçek V. (2022). Evaluating systemic immune-inflammation index in patients with implantable cardioverter defibrillator for heart failure with reduced ejection fraction. Pacing Clin. Electrophysiol..

[16] Yuan M., Ren F., Gao D. (2022). The value of SII in predicting the mortality of patients with heart failure. Dis. Markers.

[17] Özmen M., Altınkaya O., Saraç İ., Aydemir S., Aydın S.Ş., Aydınyılmaz F., Aksakal E. (2025). Prognostic impact of systemic immune inflammatory index in patients with reduced ejection fraction heart failure. BMC Cardiovasc. Disord..

[18] Yang X., Tao N., Wang T., Zhang Z., Wu Q. (2025). The relationship between composite inflammatory indicators and short-term outcomes in patients with heart failure. Int. J. Cardiol..

[19] Yu J., Zuo T., Peng S., Xu D. (2025). Diagnostic and prognostic value of systemic immune-inflammation index for heart failure: A systematic review and meta-analysis. Front. Cardiovasc. Med..

[20] Zhu S., Li H., Ou Z., Zheng M., Yuan W. (2026). Systemic immune-inflammation index outperforms conventional inflammatory markers in predicting cardiovascular outcomes in heart failure with preserved ejection fraction. J. Inflamm. Res..

[21] Zhang J., Lyu N., Zhang Y., Chen P., Ma Q., Hu N., Men B., Shi X., Wang C., Zhang J. (2026). Association between systemic immune-inflammation index (SII) and all-cause and cardiovascular mortality in heart failure patients: A single-center retrospective analysis. Front. Cardiovasc. Med..

