# Supplementary material for: Beyond Ejection Fraction: Inflammatory Risk and Mortality in Heart Failure
Source: Clin Pract. 2026 Sep 22;16(10):175. doi: 10.3390/clinpract16100175 (PMC13703748; doi:10.3390/clinpract16100175)
Supplement: Supplementary file 1 [file clinpract-16-00175-s001.zip › Supplementary1.pdf]

Supplementary Table S1. Calibration and internal validation of the clinical and laboratory model for recorded all-cause mortality

Calibration measure	Estimate
Patients included, n	634
Recorded deaths, n (%)	165 (26.0%)
Mean predicted probability	0.260
Hosmer-Lemeshow chi-square (df)	6.66 (8)
Hosmer-Lemeshow p-value	0.573
Apparent calibration intercept (95% CI)	0.00 (-0.19 to 0.19)
Apparent calibration slope (95% CI)	1.00 (0.77 to 1.23)
Bootstrap optimism-corrected calibration slope	0.82
Apparent Brier score	0.164
Bootstrap optimism-corrected Brier score	0.174

The model included age, sex, LVEF phenotype, coronary artery disease, previous myocardial infarction, previous stroke, diabetes mellitus, arterial hypertension, obesity, atrial fibrillation, dyslipidemia, log2-transformed NT-proBNP and SII, GFR, and hemoglobin. Internal validation was performed using 1,000 bootstrap samples. Apparent calibration intercept and slope are estimated in the development dataset and are therefore expected to be approximately 0 and 1, respectively. CI, confidence interval; df, degrees of freedom.
