# Supplementary material for: Beyond Ejection Fraction: Inflammatory Risk and Mortality in Heart Failure
Source: Clin Pract. 2026 Sep 22;16(10):175. doi: 10.3390/clinpract16100175 (PMC13703748; doi:10.3390/clinpract16100175)
Supplement: Supplementary file 1 [file clinpract-16-00175-s001.zip › Supplementary2.pdf]

Supplementary Table S2. Assessment of multicollinearity in the clinical and laboratory model

Predictor	VIF	Tolerance
Age (per 10 years)	1.44	0.69
Male sex	1.19	0.84
HFrEF vs HFpEF	1.42	0.70
HFmrEF vs HFpEF	1.14	0.88
CAD	1.41	0.71
Previous MI	1.36	0.74
Previous stroke	1.04	0.96
Diabetes mellitus	1.11	0.90
Arterial hypertension	1.10	0.91
Obesity	1.15	0.87
Atrial fibrillation	1.19	0.84
Dyslipidemia	1.18	0.85
NT-proBNP (per doubling)	1.66	0.60
SII (per doubling)	1.13	0.89
GFR (per 10 units)	1.27	0.79
Hemoglobin (per 10 g/L)	1.21	0.83

The maximum VIF was 1.66, and the maximum condition index was 2.34. No evidence of problematic multicollinearity was observed. VIF, variance inflation factor. Tolerance was calculated as 1/VIF.
