# Supplementary material for: Beyond Ejection Fraction: Inflammatory Risk and Mortality in Heart Failure
Source: Clin Pract. 2026 Sep 22;16(10):175. doi: 10.3390/clinpract16100175 (PMC13703748; doi:10.3390/clinpract16100175)
Supplement: Supplementary file 1 [file clinpract-16-00175-s001.zip › Supplementary3.pdf]

Supplementary Table S3. Interaction analysis between laboratory parameters and LVEF phenotype for recorded all-cause mortality

Likelihood-ratio tests comparing the fully adjusted clinical and laboratory model with the corresponding model containing the biomarker-by-phenotype interaction terms.

Interaction term	LR χ^2	df	Interaction p-value
NT-proBNP × LVEF phenotype	1.09	2	0.578
SII × LVEF phenotype	0.97	2	0.617
GFR × LVEF phenotype	2.91	2	0.234
Hemoglobin × LVEF phenotype	0.58	2	0.749

Abbreviations: LR, likelihood ratio; LVEF, left ventricular ejection fraction; NT-proBNP, N-terminal pro-B-type natriuretic peptide; SII, systemic immune-inflammation index; GFR, glomerular filtration rate.

Note: Each interaction was tested by adding two phenotype-specific interaction coefficients to the final clinical and laboratory logistic regression model. The reference phenotype was HFpEF. All analyses used the same 634 complete cases, including 165 recorded deaths. P-values are based on 2-degree-of-freedom likelihood-ratio tests.
