# Supplementary material for: Beyond Ejection Fraction: Inflammatory Risk and Mortality in Heart Failure
Source: Clin Pract. 2026 Sep 22;16(10):175. doi: 10.3390/clinpract16100175 (PMC13703748; doi:10.3390/clinpract16100175)
Supplement: Supplementary file 1 [file clinpract-16-00175-s001.zip › Supplementary4.pdf]

Supplementary Table S4. Exploratory incremental model performance after addition of SII

Model	AIC	BIC	AUC (95% CI)	Brier score
Clinical variables + NT-proBNP	680.16	742.48	0.710 (0.662-0.754)	0.170
Clinical variables + NT-proBNP + SII	665.99	732.77	0.732 (0.686-0.776)	0.164

Incremental comparison: LR chi-square=16.17, df=1, p<0.001; delta AIC=-14.17; delta BIC=-9.72; delta AUC=0.021 (bootstrap 95% CI -0.001 to 0.045), DeLong p=0.076; delta Brier score=-0.005.

Note. Both models were fitted to the same 634 complete cases (165 recorded deaths). Clinical variables included age, sex, LVEF phenotype, coronary artery disease, previous myocardial infarction, stroke, diabetes mellitus, arterial hypertension, obesity, atrial fibrillation, and dyslipidemia. NT-proBNP and SII were log2-transformed and interpreted per doubling. AUC confidence intervals and the confidence interval for the AUC difference were estimated using 2,000 stratified bootstrap resamples. AUCs were compared using DeLong's test. Lower AIC, BIC, and Brier scores indicate better model performance.
