# Supplementary material for: Beyond Ejection Fraction: Inflammatory Risk and Mortality in Heart Failure
Source: Clin Pract. 2026 Sep 22;16(10):175. doi: 10.3390/clinpract16100175 (PMC13703748; doi:10.3390/clinpract16100175)
Supplement: Supplementary file 1 [file clinpract-16-00175-s001.zip › Supplementary5.pdf]

Supplementary restricted cubic spline analysis

Supplementary Table S5. Restricted cubic spline analysis of SII and recorded all-cause mortality

Comparison	LR chi-square	df	p-value
Overall SII association (spline model vs model without SII)	21.37	3	<0.001
Non-linearity (spline model vs linear SII model)	5.98	2	0.050

Analysis details: The analysis included 634 complete cases with 165 recorded deaths. SII was log2-transformed and modeled using a restricted cubic spline with four knots at the 5th, 35th, 65th, and 95th percentiles of log2(SII). The reference value was the sample median SII (915.4). The model was adjusted for age, sex, LVEF phenotype, coronary artery disease, previous myocardial infarction, stroke, diabetes mellitus, arterial hypertension, obesity, atrial fibrillation, dyslipidemia, NT-proBNP, GFR, and hemoglobin.

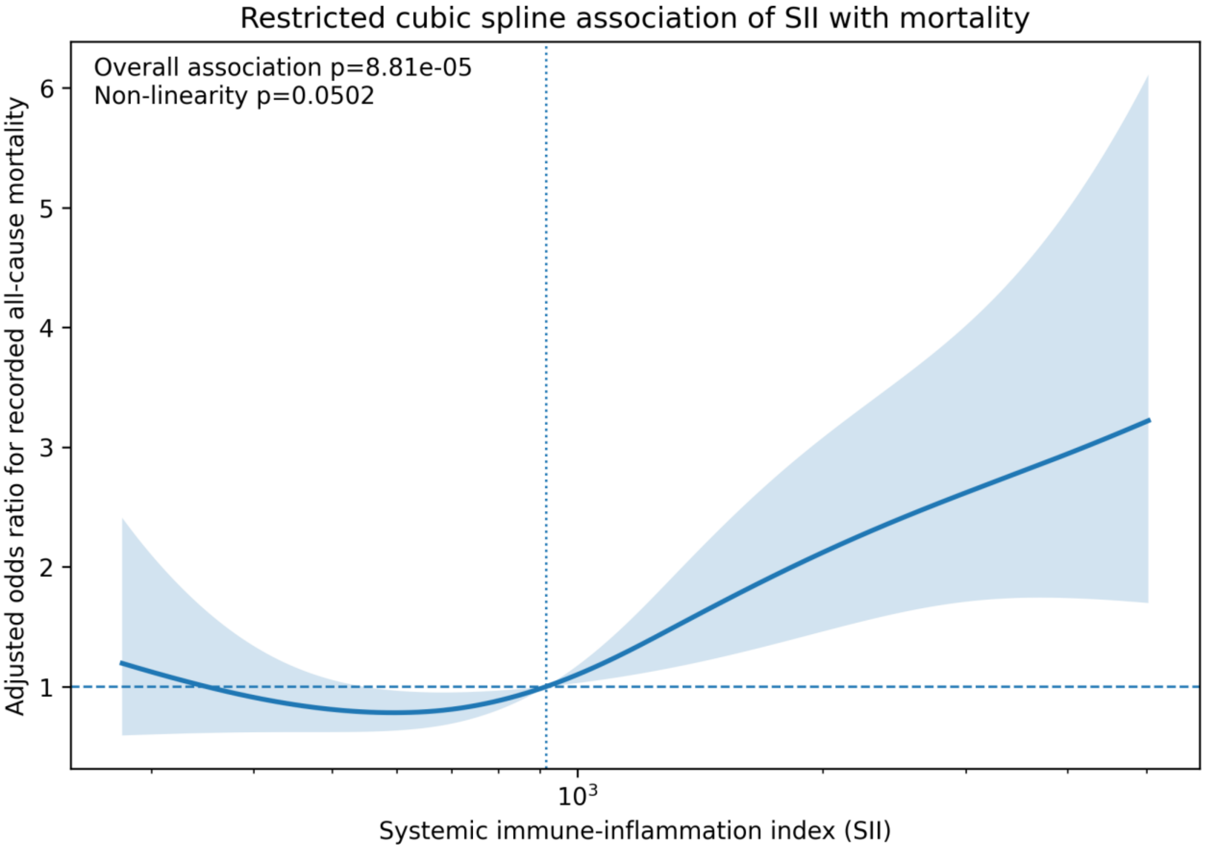

Supplementary Figure S1. Adjusted restricted cubic spline association between SII and recorded all-cause mortality. The solid line shows the adjusted odds ratio and the shaded area the 95% confidence interval. The horizontal dashed line indicates OR=1, and the vertical dotted line indicates the median SII reference value.
