# Supplementary material for: Beyond Ejection Fraction: Inflammatory Risk and Mortality in Heart Failure
Source: Clin Pract. 2026 Sep 22;16(10):175. doi: 10.3390/clinpract16100175 (PMC13703748; doi:10.3390/clinpract16100175)
Supplement: Supplementary file 1 [file clinpract-16-00175-s001.zip › Supplementary6..pdf]

Supplementary Table S6. Sensitivity analysis restricted to patients with at least 12 months of potential observation

Variable	Adjusted OR	95% CI	p-value
Age, per 10-year increase	1.22	0.97–1.53	0.086
Male sex	0.96	0.61–1.51	0.853
HFrEF vs HFpEF	0.96	0.56–1.67	0.891
HFmrEF vs HFpEF	1.63	0.91–2.93	0.103
Coronary artery disease	1.01	0.56–1.80	0.985
Previous myocardial infarction	3.02	1.51–6.04	0.002
Previous stroke	1.55	0.87–2.77	0.136
Diabetes mellitus	1.07	0.67–1.70	0.771
Arterial hypertension	0.63	0.36–1.08	0.092
Obesity	0.65	0.27–1.55	0.331
Atrial fibrillation	1.72	1.10–2.70	0.018
Dyslipidaemia	0.77	0.48–1.23	0.272
NT-proBNP, per doubling	1.24	1.08–1.43	0.003
SII, per doubling	1.48	1.22–1.80	<0.001
eGFR, per 10 mL/min/1.73 m² increase	0.99	0.90–1.08	0.760
Haemoglobin, per 10 g/L increase	0.94	0.85–1.03	0.175

Sensitivity cohort: n=549, including 147 deaths. Model −2 Log Likelihood=541.146; AIC=575.146.

Primary-analysis comparison: SII OR 1.42 (95% CI 1.19–1.70; p<0.001) in n=634 versus OR 1.48 (95% CI 1.22–1.80; p<0.001) in the ≥12-month potential-observation sensitivity cohort.

Mortality status was ascertained on 1 July 2026. The sensitivity cohort therefore included patients whose index hospitalization occurred on or before 1 July 2025. The fully adjusted logistic regression model was repeated using the same covariates as in the primary analysis. This sensitivity analysis does not replace time-to-event analysis.

Abbreviations: CI, confidence interval; eGFR, estimated glomerular filtration rate; HFmrEF, heart failure with mildly reduced ejection fraction; HFpEF, heart failure with preserved ejection fraction; HFrEF, heart failure with reduced ejection fraction; NT-proBNP, N-terminal pro-B-type natriuretic peptide; OR, odds ratio; SII, systemic immune-inflammation index.
