# Supplementary material for: Beyond Ejection Fraction: Inflammatory Risk and Mortality in Heart Failure
Source: Clin Pract. 2026 Sep 22;16(10):175. doi: 10.3390/clinpract16100175 (PMC13703748; doi:10.3390/clinpract16100175)
Supplement: Supplementary file 1 [file clinpract-16-00175-s001.zip › Supplementary7.pdf]

Supplementary Table S7. Sensitivity analysis using the 2021 CKD-EPI creatinine equation in the fully adjusted mortality model.

Variable	Adjusted OR	95% CI	p-value
Age, per 10-year increase	1.17	0.95–1.44	0.140
Male sex	1.02	0.67–1.55	0.933
HFrEF vs HFpEF	0.81	0.49–1.35	0.424
HFmrEF vs HFpEF	1.53	0.89–2.63	0.125
Coronary artery disease	0.92	0.54–1.55	0.744
Previous myocardial infarction	2.76	1.49–5.11	0.001
Previous stroke	1.61	0.95–2.72	0.075
Diabetes mellitus	1.12	0.73–1.70	0.614
Arterial hypertension	0.68	0.41–1.13	0.136
Obesity	0.64	0.29–1.41	0.263
Atrial fibrillation	1.63	1.08–2.47	0.021
Dyslipidaemia	0.75	0.49–1.16	0.197
NT-proBNP, per doubling	1.22	1.08–1.39	0.002
SII, per doubling	1.43	1.19–1.71	<0.001
CKD-EPI 2021 eGFR, per 10 mL/min/1.73 m^2 increase	0.98	0.90–1.06	0.582
Haemoglobin, per 10 g/L increase	0.97	0.89–1.05	0.423

Data are from the same 634-patient complete-case sample used for the primary mortality analysis. eGFR was recalculated using the 2021 CKD-EPI creatinine equation and substituted for MDRD eGFR in the fully adjusted logistic regression model. NT-proBNP and SII were $\log_2$-transformed. Reference categories were female sex and HFpEF. OR, odds ratio; CI, confidence interval; eGFR, estimated glomerular filtration rate; HFrEF, heart failure with reduced ejection fraction; HFmrEF, heart failure with mildly reduced ejection fraction; HFpEF, heart failure with preserved ejection fraction; SII, systemic immune-inflammation index.

Model fit: −2 Log Likelihood = 634.963; AIC = 668.963.
