# Supplementary material for: Beyond Ejection Fraction: Inflammatory Risk and Mortality in Heart Failure
Source: Clin Pract. 2026 Sep 22;16(10):175. doi: 10.3390/clinpract16100175 (PMC13703748; doi:10.3390/clinpract16100175)
Supplement: Supplementary file 1 [file clinpract-16-00175-s001.zip › Supplementary8.pdf]

Supplementary Table S8. Sensitivity analysis including CRP in the fully adjusted mortality model

Variable	Adjusted OR	95% CI	p-value
Age, per 10-year increase	1.21	0.98–1.50	0.078
Male sex	0.98	0.63–1.51	0.916
HFrEF vs HFpEF	0.96	0.57–1.62	0.893
HFmrEF vs HFpEF	1.59	0.91–2.80	0.106
Coronary artery disease	0.97	0.56–1.67	0.910
Previous myocardial infarction	2.69	1.41–5.12	0.003
Previous stroke	1.50	0.87–2.58	0.140
Diabetes mellitus	1.12	0.72–1.75	0.607
Arterial hypertension	0.64	0.38–1.07	0.090
Obesity	0.62	0.28–1.39	0.246
Atrial fibrillation	1.57	1.02–2.41	0.041
Dyslipidaemia	0.75	0.48–1.18	0.216
NT-proBNP, per doubling	1.15	1.01–1.32	0.034
SII, per doubling	1.26	1.04–1.53	0.021
eGFR, per 10 mL/min/1.73 m^2 increase	0.98	0.91–1.07	0.694
Haemoglobin, per 10 g/L increase	0.94	0.86–1.03	0.205
CRP, per doubling	1.17	1.04–1.32	0.010

Complete-case cohort: n=573, including 157 deaths. Model −2 Log Likelihood=588.230; AIC=624.230.

The fully adjusted logistic regression model was repeated among 573 complete cases with available mortality status and CRP data, with log_2-transformed CRP added as an additional covariate.

Reference categories: female sex and HFpEF. NT-proBNP, SII, and CRP are reported per doubling; age, eGFR, and haemoglobin are reported per the increments shown.

Abbreviations: CI, confidence interval; CRP, C-reactive protein; eGFR, estimated glomerular filtration rate; HFmrEF, heart failure with mildly reduced ejection fraction; HFpEF, heart failure with preserved ejection fraction; HFrEF, heart failure with reduced ejection fraction; NT-proBNP, N-terminal pro-B-type natriuretic peptide; OR, odds ratio; SII, systemic immune-inflammation index.
